# Artificial intelligence in public health education: current evidence, competencies, and a framework for responsible integration

**DOI:** 10.3389/fpubh.2026.1951054

**Published:** 2026-09-15

**Authors:** Hisham Arab, Ghazal Assaad Mirdad, Maha Abulfetoh Mohamed Abd-Allah

**Affiliations:** 1 Women and Fetal Health Program, Dr. Arab Medical Center, Jeddah, Saudi Arabia; 2 Department of Preventive Medicine and Public Health, King Abdulaziz University, Jeddah, Saudi Arabia; 3 Evidence for Clinical Research Solutions, Cairo, Egypt

**Keywords:** AI literacy, artificial intelligence, curriculum development, ethical AI, generative AI, public health education

## Abstract

Public health practice and education are being profoundly transformed by artificial intelligence (AI), yet formal AI literacy training in academic programs remains lacking. This narrative review synthesizes literature identified through a structured search of PubMed, Scopus, Web of Science, and Google Scholar for publications issued between January 2020 and June 2026. The narrative synthesis draws on 21 publications, including empirical studies, reviews, perspectives, educational frameworks, and authoritative guidance documents. The review underscores a significant gap between the widespread use of informal AI and the limited availability of formal curricula, with most evidence drawn from medical education rather than public health-specific settings. Key applications include adaptive learning platforms, AI-enhanced simulation, and research training support. We propose the Public Health AI–Education Integration Framework comprising nine domains: learner and curriculum needs assessment; AI literacy foundation; AI tool selection and educational alignment; public health dataset and case integration; adaptive learning and simulation delivery; human-in-the-loop supervision; bias, equity, and ethical auditing; assessment and academic integrity; and feedback, monitoring, and curriculum refinement. The framework is proposed as a conceptual model requiring prospective validation, intended to move beyond *ad hoc* AI use toward structured, competency-based training that strengthens analytical reasoning, ethical judgment, and equity-oriented practice in AI-enabled public health systems.

## 1 Introduction

Artificial intelligence is transforming nearly every facet of public health, from disease surveillance to policy development. Large language models such as ChatGPT, Claude, DeepSeek, Llama, Copilot, and Gemini are now employed in epidemiological modeling, crisis response, and health communication, offering powerful new tools for data-driven decision-making. In public health, the use of artificial intelligence (AI) is being explored for disease surveillance, forecasting disease outbreaks, risk stratification, health communication and misinformation detection, resource allocation, evidence synthesis, and policy modeling (1–3). These developments are occurring in parallel with a second transition in higher education, where generative AI tools are being used for writing support, tutoring, assessment preparation, literature summarization, coding support, and feedback generation (4–6). Public health education sits at the nexus of these two transitions. Public health graduates will need to work in environments increasingly populated by digital platforms, large datasets, predictive models, dashboards, and AI-supported decision systems (1–3). But many students and educators are still learning about AI through informal experimentation instead of through a structured curriculum. This creates a gap between the technological realities of public health practice and the competencies formally taught in public health programs. Recent literature has emphasized that AI literacy should become a core component of public health education (1). AI literacy in this context does not mean training every student to become a machine-learning engineer. Rather, it refers to the ability to understand what AI systems can and cannot do, to critically evaluate AI outputs, to recognize bias and uncertainty, to protect privacy, to communicate limitations, and to use AI responsibly in public health decision-making (1–3, 7). Recognizing the significance of AI in advancing global health objectives, the World Health Organization has highlighted the need for AI literacy as a core competency for the next generation of healthcare professionals (7). Despite growing interest, existing reviews have predominantly focused on medical education or general health professions education, with limited attention to the population-level, policy-oriented, and equity-focused dimensions that distinguish public health practice. This review therefore addresses a specific gap by synthesizing evidence specifically relevant to public health education, identifying competencies unique to the discipline, and proposing a structured framework for responsible AI integration. This review therefore aims to: (1) synthesize current trends in AI use in public health teaching and education; (2) summarize comparative evidence from recent studies and frameworks; and (3) propose a structured model for responsible integration of AI into public health curricula.

## 2 Literature search and study selection

Given the narrative nature of this review, a structured literature search was conducted to identify relevant publications. PubMed, Scopus, Web of Science, and Google Scholar were searched for English-language publications from January 2020 to June 2026. The search was last conducted on 30 June 2026. This period was selected to capture recent developments in AI-assisted education, including those following the public release of ChatGPT in November 2022 and the accelerated digital transformation of education during and after the COVID-19 pandemic.

### 2.1 Search strategy

The search strategy combined terms related to artificial intelligence with terms related to public health education, competencies, curricula, and critical appraisal. The search syntax was adapted to the requirements of each database. The exact search strategies and numbers of records retrieved were as follows.

PubMed (*n* = 11).

((“public health education”[tiab] OR “public health students”[tiab]) AND (“AI literacy”[tiab] OR “generative AI”[tiab] OR “artificial intelligence”[tiab] OR “large language model*”[tiab] OR ChatGPT[tiab] OR “machine learning”[tiab]) AND (competenc*[tiab] OR framework*[tiab] OR curricul*[tiab] OR “skill development”[tiab] OR “critical appraisal”[tiab]))

Filter applied: publication date from January 2020 to June 2026.

Google Scholar (*n* = 85).

(“public health education” OR “public health students”) AND (“artificial intelligence” OR “generative AI” OR “large language models” OR ChatGPT OR “machine learning”) AND (competency OR competencies OR curriculum OR assessment OR “critical appraisal” OR framework)

Filter applied: publication date from January 2020 to June 2026.

Web of Science (*n* = 22).

TS = ((“public health education” OR “public health students”) AND (“AI literacy” OR “generative AI” OR “artificial intelligence” OR “large language model*” OR ChatGPT OR “machine learning”) AND (competenc* OR framework* OR curricul* OR “skill development” OR “critical appraisal”)).

Filter applied: publication date from January 2020 to June 2026.

Scopus (*n* = 15).

TITLE-ABS-KEY((“public health education” OR “public health students”) AND (“AI literacy” OR “generative AI” OR “artificial intelligence” OR “large language model*” OR ChatGPT OR “machine learning”) AND (competenc* OR framework* OR curricul* OR “skill development” OR “critical appraisal”)).

Filter applied: publication date from January 2020 to June 2026.

### 2.2 Eligibility criteria

Eligible publications included empirical studies, systematic and scoping reviews, educational frameworks, perspectives, commentaries, and authoritative guidance documents addressing AI applications, AI-related competencies, governance, assessment, or curriculum development in public health education or closely related health-professions education.

Publications were eligible if they: (1) addressed the use or integration of AI in an educational context; (2) focused on public health education or provided findings or recommendations transferable to public health from health informatics, medical education, or broader health-professions education; (3) examined AI-related teaching, learning, assessment, competencies, curriculum development, governance, ethics, or equity; and (4) were published in English between January 2020 and 30 June 2026.

Publications were excluded if they: (1) did not address an educational context; (2) focused exclusively on technical AI development or clinical performance without educational relevance; (3) did not provide findings, frameworks, or recommendations applicable to public health or health-professions education; or (4) did not have an accessible full text.

### 2.3 Screening and publication selection

The database searches identified 133 records: 11 from PubMed, 85 from Google Scholar, 22 from Web of Science, and 15 from Scopus. After 21 duplicate records were removed, 112 unique records remained for screening.

Two reviewers independently screened the titles and abstracts of the 112 records against the eligibility criteria. Disagreements were resolved through discussion and consensus. Following title and abstract screening, 78 records were excluded, and the full texts of the remaining 34 records were sought. Fourteen full texts could not be retrieved, leaving 20 publications for full-text eligibility assessment.

The two reviewers independently assessed the 20 full-text publications against the eligibility criteria. Sixteen publications were excluded: nine because their focus was not relevant to the review objectives and seven because their publication type or design did not meet the eligibility criteria. Consequently, four publications identified through the database searches were included.

Backward and forward citation searching of the eligible publications and relevant reviews was subsequently undertaken to identify additional sources. This process identified 17 additional publications, which were assessed against the same eligibility criteria. In total, 21 publications were included in the narrative synthesis.

The included literature comprised empirical educational studies, systematic and scoping reviews, perspectives, educational and competency frameworks, and policy or governance documents. Given the narrative nature of the review and the heterogeneity of the included publications, no formal risk-of-bias assessment or quantitative synthesis was undertaken.

## 3 Narrative synthesis of current evidence

### 3.1 Overview of the selected literature

The narrative synthesis was conducted using 21 selected publications on AI in public health and health professions education. The literature selected comprised empirical educational studies, systematic and scoping reviews, perspective and framework papers, and policy or governance documents. Importantly, the evidence base for public health education is relatively small, and a significant proportion of it is drawn from medical education, health informatics, and general health professions education. Key themes discussed were the development of teaching and learning approaches with AI-powered simulations and customized learning environments, the expansion of AI and online literacy education, varying levels of institutional readiness, and urgent ethical concerns about algorithmic fairness and inclusion. Interdisciplinary collaboration and curriculum reform were identified as critical to maintaining AI integration.

### 3.2 Current trends in AI use in public health teaching

AI is being incorporated into public health and health professions education through several primary mechanisms. The most visible trend is the use of generative AI tools as learning assistants, where students use these tools to summarize readings, clarify concepts, generate quiz questions, draft outlines, translate technical information, and receive immediate explanations, while faculty use similar tools to create teaching materials, case scenarios, rubrics, formative assessment items, and feedback prompts (8). Generative AI models are increasingly used to answer student queries, create personalized lessons, provide tailored feedback, and even simulate virtual patients based on aggregated data (9). A second notable trend involves the move toward adaptive and personalized learning environments, in which AI-driven systems use student performance data to pinpoint knowledge gaps, modulate content difficulty, suggest supplementary resources, and deliver real-time feedback (2). Wang and colleagues highlighted opportunities for AI in individualized instruction, behavioral modeling, and public health policy applications, capabilities especially valuable for quantitatively demanding disciplines such as epidemiology, biostatistics, disease modeling, and health informatics (2). A third emerging trend is the integration of AI into scenario-based and simulation-based learning, enabling dynamic scenarios in which students respond to evolving outbreak conditions, communication challenges, policy constraints, or resource allocation problems (2, 3). These AI-enhanced simulations adapt in real time to learner inputs, offering a personalized experience that mirrors the complexity of real-world public health practice (10). They include simulations of disease outbreaks, exercises in vaccine hesitancy, emergency preparedness drills, assessments of occupational hazards, and evaluations of community needs (3). Patil et al. (11) reported high levels of engagement with an AI-augmented simulation activity among undergraduate public health students, while Howard (10) described AI-driven outbreak simulations that immerse students in realistic, high-pressure situations to develop critical crisis communication skills. However, these findings are primarily based on student-reported measures rather than objective learning outcomes, and evidence of sustained educational benefit remains limited. Comulada et al. (12) found that incorporating AI-based digital health interventions into graduate curricula elicited positive student responses, although participants raised concerns about their relevance to more traditional public health practice. A fourth trend is AI-assisted research training, where AI can support literature reviews, evidence synthesis, research proposals, data analysis, and scientific writing, but only when students are trained to verify outputs, detect hallucinated references, evaluate source quality, and document AI assistance transparently (1, 5, 7, 9). Finally, there is growing attention to institutional governance, as universities and schools of public health are beginning to develop policies on acceptable AI use, academic integrity, privacy, assessment design, and disclosure, though guidance remains variable and public health-specific frameworks are still emerging (1, 13).

A critical observation across these trends is that while AI applications are proliferating, the empirical evidence evaluating their educational effectiveness in public health contexts remains limited. A 2026 systematic review restricted to randomized controlled trials found insufficient evidence to establish a consistent educational benefit. Heterogeneity, imprecision, and a lack of higher-level outcomes limited the conclusions (14). This suggests that current trends should be characterized as experimental and promising rather than established and validated.

### 3.3 Comparative evidence from recent studies

Table 1 presents a comparative synthesis of studies and tools relevant to AI in public health education. Because direct public health education studies remain limited, the table includes three categories: public health education studies (Direct), health informatics/public health-adjacent education studies (Adjacent), and broader health professions education studies with transferable implications (Indirect).

**Table 1:** Comparative evidence on AI tools and studies relevant to public health education.

Main author/year	Setting/population	AI tool/approach	Purpose for education	Key findings and contribution	Relevance to public health education	Evidence directness
Acosta (1)	Public health education perspective	AI literacy framework	Curriculum integration	Advocates for incorporating AI literacy across levels of public health education, with attention to ethical understanding, critical evaluation, and technical proficiency.	Provides a rationale for integrating AI literacy into public health curricula and identifies ethical and technical competencies to inform curriculum development.	Direct
Wang et al. (2)	Public health education review	AI, big data, behavioral modeling, personalized education	Public health teaching and policy-oriented learning	AI’s use for individualized instruction, behavioral interventions, and policy analysis, tying AI tools to specific public health disciplines rather than treating AI as a generic add-on.	The review supports integrating AI applications into existing public health courses, although its broad scope limits detailed evaluation of individual educational applications.	Direct
Gordon et al. (17)	Multi-national scoping review	Multiple AI technologies	Teaching, assessment, clinical reasoning	The review synthesizes evidence across educational settings and proposes a classification framework for categorizing AI tools according to their intended educational purpose.	Though developed for medical education, the classification methods transfer well to public health contexts. Public health programs could adopt this framework with minimal adaptation.	Indirect
Feigerlova et al. (16)	Health professions education systematic review	AI-powered educational interventions	Educational outcome evaluation	The evidence supporting AI-powered educational interventions remains limited. Few studies assessed objective learning outcomes or employed rigorous comparative designs, underscoring the need for controlled, longitudinal evaluations.	The findings highlight the need for rigorous, controlled, and longitudinal evaluations of AI-supported educational interventions before their effectiveness can be established.	Indirect
Parmar et al. (3)	Postgraduate public health students in India	ChatGPT-based blended teaching intervention	Occupational health teaching	Combined ChatGPT with field exposure, expert input, and student presentations. The approach was well received, indicating that it is feasible and acceptable from students’ perspective to integrate generative AI into practice-based teaching.	The study will be of value to educators seeking a concrete, replicable model. It demonstrates how AI can augment, rather than replace, practical learning.	Direct
Hunt et al. (20)	Undergraduate medical education	Multiple AI competencies	Competency framework development	The review analyzed more than 500 competency statements and synthesized them into seven domains, 37 competencies, and 170 learning objectives.	Provides a ready-made structure for institutions developing new AI curricula. The domains of ethics, law, professionalism, critical appraisal, and research are directly transferable to public health.	Indirect
Seba et al. (4)	Graduate health informatics students	Generative AI curriculum module	Health Informatics competencies	The dedicated generative AI module helped students feel more confident and competent to use AI tools. The pre-post design adds to the credibility of the findings, but a lack of a control group is a limitation to note.	This is relevant, as health informatics is becoming increasingly central to public health practice. It also provides lessons for structuring AI content in other public health disciplines.	Adjacent
Conrad and Hall (15)	Public health and health promotion education	Generative AI	Curriculum design and pedagogy	Explains how generative AI can improve curriculum development, teaching strategies, assessment, feedback, and DEI efforts. Most importantly, capacity-building around AI is a prerequisite to improving the quality of education, not an afterthought.	Speaks directly to the faculty development challenge. Institutions need to do more than buy AI tools; they need to train educators to use them well. This is a message that often gets overlooked.	Direct
Wang et al. (7)	Global health governance guidance	Large multimodal models	Ethics and governance	This contribution draws from WHO guidance and offers practical recommendations for the safe and equitable use of large multimodal AI models. The emphasis on transparency, bias mitigation, and health equity is particularly valuable for educators and practitioners.	The publication provides recommendations relevant to teaching the ethical and governance dimensions of AI, including transparency, bias mitigation, and health equity.	Adjacent
Miao and Holmes et al. (19)	Global education and research guidance	Generative AI	Education policy and governance	UNESCO’s guidance also advises on policy development, regulation, human capacity building and frameworks for responsible use. This focus on institutional governance is especially pertinent as schools of public health navigate this rapidly changing terrain.	A useful resource for administrators and curriculum committees to help them design institutional policies for AI use, academic integrity, and data governance.	Adjacent
Astbury et al. (8)	Health professions education scoping review	Generative AI	Teaching and assessment	This scoping review examined generative AI across multiple health professions and proposed a practical checklist to support its responsible integration into teaching and assessment.	For educational programs moving from policy to practice, the proposed checklist offers a structured approach for integrating generative AI responsibly into teaching and assessment.	Indirect
Dankwa-Mullan (22)	Public health and medicine commentary	Multiple AI technologies	Health equity and ethics	Argues for a multi-pronged strategy that integrates data governance, ethical frameworks, community engagement, education, and continuous assessment. The focus on interconnectedness is important as AI equity cannot be tackled in isolation.	It emphasizes that AI is not only a technical tool but also a sociotechnical intervention. Public health educators should infuse this perspective into the curriculum, rather than confine it to a stand-alone ethics course.	Direct
Abeo et al. (21)	Health literacy education	Multiple AI technologies	Health literacy improvement	The purpose of this systematic review is to examine the use of AI in health literacy and to point out its opportunities and limitations. AI can improve readability, but further research is needed to ensure completeness and cultural appropriateness, a finding with important practical implications.	It is useful for health communication training and for preparing students to critically assess AI-generated content. Also highlights the importance of cultural adaptation, which deserves more attention in the literature.	Indirect
Ormeño Rivera et al. (23)	Health science education systematic review	Multiple AI technologies	Teacher training	The review concluded that the educational use of AI requires faculty preparation addressing both technical and ethical considerations and identified faculty development as an important component of institutional planning.	Directly addresses the faculty development challenge. The more important investment is in teacher training, rather than in AI tools themselves.	Indirect
Patil et al. (11)	Undergraduate public health students	AI-enhanced simulation (ChatGPT, Adobe Firefly)	Global health education	The Global Health Trade Summit simulation used AI to create an engaging learning experience. Students reported high levels of engagement and increased understanding of international trade concepts. The simulation incorporated multiple AI tools, including ChatGPT and Adobe Firefly.	Provides a scalable model for public health courses seeking to integrate experiential AI education, particularly for global health and policy content.	Direct
Howard et al. (10)	Undergraduate and postgraduate public health students	AI-driven outbreak simulations powered by LLMs	Outbreak management training	Developed realistic, high-pressure outbreak scenarios that challenge students to build crisis communication and decision-making skills. The simulation incorporated multiple AI tools, including ChatGPT and Adobe Firefly.	Directly applicable to emergency preparedness and outbreak response training. Such a simulation could also be particularly useful for programs training public health professionals for the field.	Direct
Hallquist et al. (9)	Medical education systematic review	Multiple AI technologies	Knowledge assessment and learning	Identifies how AI is being used to generate exam questions, clinical scripts, and diagnostic exercises. The review identified potential applications in question generation, clinical scripts, diagnostic exercises, and formative assessment, although evidence of effectiveness and scalability remains limited.	Demonstrates how AI could support assessment and feedback in ways that could be adapted for public health. The implications for large classes and resource-constrained settings are significant.	Indirect
Comulada et al. (12)	Graduate public health and clinical students	Chatbots, VR/AR, AI technologies	Digital health intervention development	Students responded favorably to AI-generated digital health content; however, many questioned its relevance to core public health practice, highlighting the importance of explicitly linking AI activities to public health competencies.	The findings indicate that AI-supported educational activities should be explicitly aligned with real-world public health competencies to enhance their perceived relevance.	Direct
Reddy (6)	Healthcare implementation science	Generative AI	Application, integration, governance	Discusses practical issues around implementation, privacy, security, and the need for human oversight. Such a focus on the practice of science offers a useful perspective often lacking in discussions of education.	Provides a useful lens for public health programs planning to incorporate AI. The emphasis on real-world implementation challenges is useful for bridging the theory-practice gap.	Indirect
Joshi et al. (13)	ASPPH member schools and programs	Generative AI policies	Institutional policy analysis	A survey of AI policies at US public health schools showed wide variation, with many lacking clear guidance. This is a significant gap, because institutions are being asked to integrate AI without having policies in place.	Reinforces the importance of developing structured institutional policies. Also suggests that schools of public health could benefit from sharing best practices and learning from each other.	Direct
Kung et al. (24)	Medical licensing examination	ChatGPT	Knowledge assessment	Reported that ChatGPT performed close to the passing threshold on the USMLE. This is a remarkable finding that demonstrates the current capabilities of large language models in health knowledge domains.	Has implications for both assessment practices and curriculum design. If AI models can pass licensing exams, it raises the question of what we are assessing and how we are assessing it a conversation public health educators need to be having.	Indirect

Evidence directness: Direct = public health education studies; Adjacent = health informatics or public health-adjacent education; Indirect = health professions education (medical/nursing/allied health) with transferable implications.

### 3.4 Required AI competencies for public health students

Public health students require a progressive, level-appropriate set of AI competencies. At the foundational level, they should understand basic AI terminology, common tool types, the generative-predictive distinction, and the limitations of AI outputs, including recognizing hallucinations, unsupported claims, and fabricated references (1, 7, 15). Medical education frameworks similarly emphasize foundational knowledge of AI and data (16). At the applied level, students should use AI for public health tasks like data exploration, communication drafting, literature searching, code support, and scenario analysis (2, 3), while also verifying outputs, explaining assumptions, and documenting AI use (7, 15). The blended pedagogical intervention described by Parmar et al. demonstrated that students can effectively use ChatGPT to explore occupational hazards and prevention strategies and to critically analyze AI-generated content (3). At the critical appraisal level, students should be able to evaluate AI tools and AI-based studies by asking whether a model was validated, which population was used for training, whether performance differs across subgroups, whether the model is interpretable, and whether the tool is appropriate for the intended public health context (1, 7, 17). Critical appraisal of AI output has been identified as a recurring area of emphasis in competency frameworks (16).

AI Literacy for Epidemiological Reasoning.

A critical gap in existing competency frameworks is insufficient attention to the statistical and data literacy needed for responsible AI use in public health. Students must understand fundamental concepts in data science and epidemiology to evaluate AI systems critically. These include training, validation, and test data; overfitting; calibration; discrimination; sensitivity and specificity; predictive values; dataset shift; external validation; missing data; representativeness; and the distinction between causal and predictive modeling.

In addition, public health students should be educated that AI-generated summaries easily blur the line between association and causation. Students should understand confounding, selection bias, collider bias, ecological fallacy, reverse causality, and the distinction between causal and predictive models.

A 2025 narrative review on AI (18) found that students could critically evaluate AI-generated language but not the model itself unless they received explicit training in data quality and model evaluation, highlighting the need for statistical literacy in AI education.

Ethically and in terms of governance, students need to know about concepts like privacy, consent, data protection, accountability, transparency, bias, health equity, and digital exclusion (7, 15), and be taught to see AI not just as a technical tool but as a sociotechnical intervention that may affect population health, institutional trust and resource allocation, with ethical and legal oversight being acknowledged as a core domain in AI competency frameworks (16). Finally, students need to be trained to work with AI, including prompt writing, critical verification, transparent disclosure, and responsible use, all without sacrificing independent reasoning (1, 8, 15).

The competency domains proposed are adapted from existing AI competency frameworks in the health professions education literature. Specifically, the seven domains proposed by Hunt et al. (2026) (16) are: AI ethics, law and regulation, professionalism, clinical applications, critical appraisal, research and innovation, and theory and foundations.

### 3.5 Ethical, legal, and equity considerations

Because public health decisions affect entire communities rather than just individuals, public health education has a distinct obligation to address the ethical dimensions of AI. AI systems can replicate or worsen inequalities if they are built on biased data, used without local validation, or deployed in environments with unequal digital access (7, 15). This is why AI integration must be grounded in a commitment to equity, institutional capacity, and ethical responsibility (19). Privacy and data protection are non-negotiable; public health datasets often include sensitive health, demographic, or geographic information, so students must learn not to upload identifiable data into unsecured platforms (7, 15), and institutions need to provide clear guidance on approved tools and data governance (5, 13). Transparency should also be a core expectation: any use of AI in coursework, teaching, analysis, or writing ought to be disclosed as a matter of responsible practice, not as a punitive control (5, 7, 15). Equity should be embedded throughout, with students gaining an understanding of how algorithms may vary in performance across different age groups, languages, ethnicities, socioeconomic backgrounds, geographic regions, and healthcare settings (1, 7). In short, ethical and equitable AI implementation requires a comprehensive approach that integrates data governance, ethical reflection, community involvement, education, and continuous monitoring (19).

A more critical discussion of governance challenges is warranted. Key implementation challenges include accountability when AI systems produce harmful outputs; the tension between transparency and intellectual property protection; algorithmic bias that may disproportionately affect marginalized populations; and the digital divide that may exacerbate existing health inequities across diverse healthcare and educational settings. These governance challenges require institutional policies that are regularly updated to keep pace with rapidly evolving AI capabilities.

### 3.6 Challenges in integrating AI into public health education

Several barriers hinder the responsible integration of AI into public health education. The most pressing of these is faculty readiness: many educators will be expected to guide students in using AI without having received much, if any, formal training themselves (1, 4, 20). This speaks to the need for faculty development that emphasizes ethical considerations, practical skills, and the development of critical and reflective thinking among teachers and students alike (21). At the same time, curricula are already dense, and programs will need to incorporate AI into existing courses without displacing core topics like epidemiology, biostatistics, health policy, social determinants, ethics, and community engagement (1). Institutional policies provide another source of inconsistency: some institutions actively promote experimentation and innovation, while others clamp down on the use of AI tools for academic integrity reasons (5, 13). The challenge is in striking a balance that allows for responsible use without undermining the educational process. There is also the risk that AI tools will exacerbate inequities if students are required to pay for premium access or if institutions are unable to provide secure, reliable platforms (7, 15). Finally, deskilling is a real concern; students may become too reliant on AI for writing, coding, analysis, or even reasoning (5, 15). This is of particular concern in public health, where professionals need to manage uncertainty, weigh evidence critically, and communicate effectively in high-pressure situations.

Particular attention should be paid to practical barriers in low- and middle-income countries (LMICs) such as infrastructure limitations (e.g., unreliable internet connectivity, insufficient computing resources), faculty preparedness (e.g., limited training opportunities, language barriers), resource constraints (e.g., inability to purchase premium AI tools, lack of technical support), and implementation challenges (e.g., cultural resistance, limited institutional capacity). If not specifically tackled by capacity-building efforts and international cooperation, these barriers could exacerbate the global health equity divide.

### 3.7 Assessment and academic integrity in the AI era

AI disrupts traditional assessment methods like unsupervised essays, take-home assignments, and generic literature summaries. Rather than relying mainly on detection tools, public health educators should redesign assessment around applied reasoning, transparency, and demonstration of competence (5, 15). Suggested approaches include oral defenses, in-class tasks, reflective AI logs, local data-based policy briefs, group simulations, critical appraisal of AI outputs, and comparisons of AI-generated versus evidence-based answers. Students could submit an AI-use statement detailing the tool used, prompts, verification process, and any modifications made (5, 15). Assessment should focus on what AI cannot replace: judgment, interpretation, ethical reasoning, communication, contextualization, community awareness, and defensible decision-making (7, 15). Practical frameworks should guide responsible use of generative AI in health professions education (8).

It is important to distinguish evidence-based recommendations from expert opinion. While assessment redesign is widely recommended in conceptual literature, empirical evidence specifically evaluating these alternative assessment approaches in public health education remains limited. Longitudinal studies comparing traditional versus AI-resistant assessments are needed to validate these recommendations.

### 3.8 Proposed framework: the public health AI-education integration framework

The nine framework domains were developed through an iterative process of author discussion and consensus. Recurring themes identified across the selected literature were grouped into preliminary domains and compared with existing AI competency frameworks and established educational models. The domains were subsequently refined to reduce conceptual overlap, improve clarity, and ensure relevance to the distinctive population-level, epidemiological, ethical, and equity-oriented requirements of public health education. This process represents a narrative and concept-development approach rather than a formal Delphi procedure or an empirical framework-validation exercise. Accordingly, the resulting framework should be regarded as a proposed conceptual model requiring external review and prospective validation.

Building upon recent calls for AI literacy, existing practices in the field, the wider health professions education literature, and guidance on ethics and governance, this narrative review draws upon recurring themes identified across the selected literature to propose a structured framework for the responsible integration of AI into public health education. The proposed framework is based on nine domains, is designed as a conceptual and practical model, and requires future evaluation and validation in different educational contexts.

The framework is grounded in existing educational models, such as competency-based education and experiential learning theory. The framework embeds AI literacy within competency-based education, requiring students to develop specific AI competencies rather than simply perform AI activities. This combination of adaptive learning and simulation delivery aligns with the theory of experiential learning, which emphasizes learning through experience and reflection, and is particularly applicable to AI-augmented simulations that provide immediate, contextual feedback (Figure 1).

**Figure 1:**
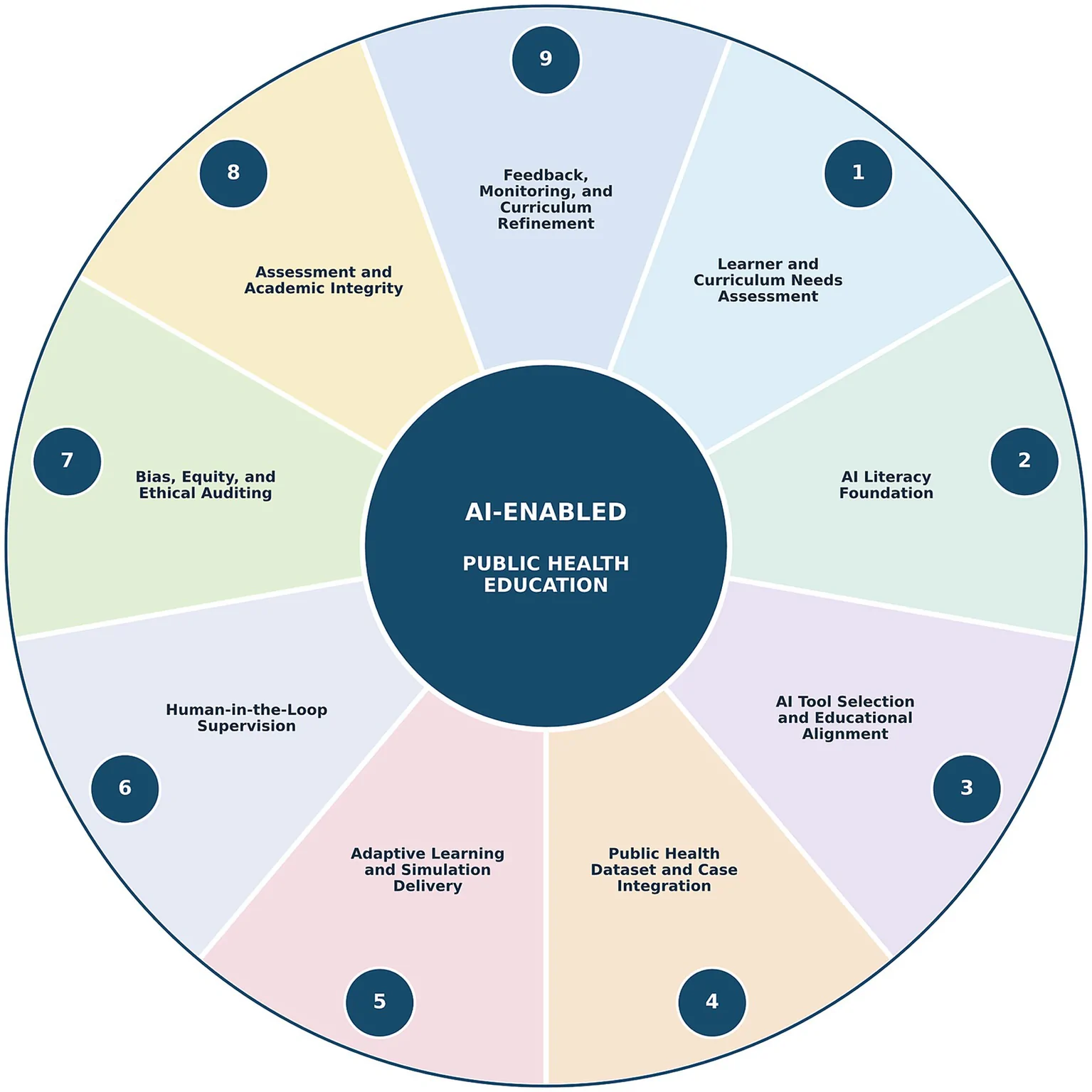
Nine-domain framework for integrating artificial intelligence into public health education. The circular framework comprises nine interrelated domains around the core goal of Artificial Intelligence-Enabled Public Health Education, which include Learner and Curriculum Needs Assessment, Artificial Intelligence Literacy Foundation, Artificial Intelligence Tool Selection and Educational Alignment, Public Health Dataset and Case Integration, Adaptive Learning and Simulation Delivery, Human-in-the-Loop Supervision, Bias, Equity, and Ethical Auditing, Assessment and Academic Integrity, and Feedback, Monitoring, and Curriculum Refinement. The circular design underscores that implementation is iterative, with feedback and monitoring driving the ongoing refinement of the curriculum.

Domain 1, Learner and Curriculum Needs Assessment, recommends that programs begin by assessing students’ current literacy in AI, quantitative skills, core public health competencies, and professional goals, and then map the curriculum to determine where AI content is most appropriate. Domain 2, AI Literacy Foundation, is designed to ensure that all students establish a solid foundation in essential AI concepts, including generative models, predictive algorithms, data quality, hallucination, bias, privacy, and ethics (1, 7, 15). This will equip learners to comprehend the limitations and strengths of AI, critically assess outputs, recognize bias and uncertainty, protect privacy, and responsibly use AI in public health work. This domain should also explicitly include components of data literacy and statistical literacy, such as training, validation, and test data; overfitting; calibration; sensitivity and specificity; predictive values; dataset shift; missing data; and the difference between causal and predictive models. Domain 3, AI Tool Selection and Educational Alignment, emphasizes the need for secure, validated, accessible, and pedagogically appropriate platforms (5, 7, 15). As Conrad and Hall (20) emphasized, capacity building in generative AI is critical for improving curriculum design, teaching approaches, assessment, and inclusivity.

Domain 4, Public Health Dataset and Case Integration, grounds learning with AI in real-world materials on surveillance data, outbreak scenarios, health promotion campaigns, policy briefs, and program evaluations (2, 3). This domain should explicitly emphasize training in causal inference, including confounding, selection bias, collider bias, ecological fallacy, reverse causality, and the distinction between prediction and explanation. Students should be trained to recognize when AI-generated summaries confuse association with causation. Domain 5, Adaptive Learning and Simulation Delivery, leverages AI to deliver personalized tutoring, immediate feedback, dynamic simulations, and scenario-based exercises tied to specific competencies (2–5, 11, 12). Domain 6, Human-in-the-Loop Supervision, preserves faculty oversight of all educational decisions, with AI supporting practice and feedback while instructors guide interpretation, assessment, and ethical reflection (7, 15).

Domain 7, Bias, Equity, and Ethical Auditing, weaves attention to bias, fairness, privacy, transparency, and population-level impacts into every AI-supported learning activity (1, 7, 15), acknowledging the interconnected nature of data governance, ethics, community engagement, education, and ongoing evaluation (19). Domain 8, Assessment and Academic Integrity, advocates for transparent AI disclosure, applied tasks, oral defenses, reflective assignments, and competency-based assessment (5, 8, 15). Finally, Domain 9, Feedback, Monitoring, and Curriculum Refinement, calls for continuous review of AI integration using student performance data, learner and faculty feedback, equity indicators, academic integrity outcomes, and relevance to evolving public health practice (15, 17). Ultimately, the framework treats AI integration as an ongoing, adaptive process rather than a one-off implementation.

Future validation should include prospective implementation studies, mixed-methods evaluations, external expert review, and longitudinal assessment of student competency development across diverse educational contexts.

## 4 Discussion

The literature synthesized in this narrative review suggests that AI is increasingly influencing public health education, although structured curricular integration and evidence of educational effectiveness remain limited. However, the evidence bases specifically evaluating AI’s educational effectiveness in public health remain much thinner than the enthusiasm surrounding the topic suggests. A considerable proportion of available literature is conceptual, opinion-based, or derived from medical education rather than public health-specific contexts. The reviewed literature consistently highlights a gap between the widespread informal use of generative AI by students and educators and the limited availability of structured curricula that guide its responsible use. Most AI applications in health professions education are currently focused on evaluating learners and programs, with additional opportunities in curriculum design, identifying learning needs, and personalizing instruction (9).

Looking across the comparative evidence, a few notable patterns stand out. Studies specifically focused on public health are relatively rare compared with the broader health professions education literature, with most of the available work coming from medical education, which may not adequately reflect the population-level, policy-oriented, and equity-focused dimensions that define public health practice. The evidence base is also quite varied, with few randomized controlled trials and limited longitudinal data (17). A 2026 systematic review/meta-analysis restricted to randomized trials concluded that the evidence remains insufficient to establish a consistent educational benefit, with heterogeneity, imprecision, and a lack of higher-level outcomes limiting the conclusions. This finding should moderate expectations about current AI integration in public health education. At the same time, ethical and governance issues are consistently highlighted across studies, signaling broad agreement that AI integration needs to be handled with care (7, 15, 19).

When the literature is classified as direct, adjacent, or indirect evidence, the analysis shows that direct evidence from public health education remains limited. Evidence from medical education and health informatics may offer transferable insights but should be applied cautiously. However, the findings from clinical education should be applied to public health at the population level with caution. The proposed framework aims to address this gap by emphasizing population-level reasoning, equity, and epidemiological thinking areas that differentiate public health from clinical education.

These observations have several important implications for health education: First, we must understand AI literacy as an emerging core competency rather than an add-on digital skill. This requires reforming the public health curriculum to embed AI throughout, rather than as a single course. Competency frameworks for health professions education have identified seven domains: AI ethics, law and regulation, professionalism, clinical applications, critical appraisal, research and innovation, and theory and foundations (16). Public health extends these by adding population-level reasoning, the distinction between causal and predictive models, and equity-oriented evaluation of AI systems. Faculty development is another priority. Many instructors lack formal training in AI and may not feel they are equipped to help students use it responsibly. Institutions should concentrate on programs that teach the technical and pedagogical aspects of AI. As Conrad and Hall (20) have argued, building capacity around generative AI is a prerequisite for improving the quality of public health and health promotion curricula. Another urgent area is assessment reform. Traditional take-home assignments are becoming vulnerable to AI abuse. Educators need to move toward assignments that emphasize applied reasoning, transparency and skills that cannot be easily replicated by AI. Finally, equity must be embedded in the integration of AI: institutions must ensure equal access to AI tools and incorporate discussions of algorithmic bias, digital exclusion and population-level equity into the educational program. While the promise of AI for public health education is great, this promise will need to be realized through careful attention to equity, institutional readiness, and ethical accountability.

However, some discrepancies are evident in the literature, as this review shows. Student satisfaction and engagement with AI-enhanced educational interventions are often reported positively, but objective learning outcomes are rarely measured, and the evidence is mixed when they are. Few studies in the systematic review by Feigerlova et al. (17) examined objective learning outcomes or used rigorous comparative designs. This implies that the current enthusiasm for AI in education is getting ahead of the evidence base. Future research should involve controlled evaluations with validated outcome measures.

AI-enabled public health education is particularly relevant to regions undergoing digital health transformation, such as the Middle East, where ongoing investments in health data systems, population health management, and workforce development create a strong need for professionals who can work with digital surveillance, predictive analytics, and AI-supported decision systems. Arabic-language AI tools and culturally adapted health communication deserve specific attention. Public health students should learn how to assess whether AI-generated Arabic health content is accurate, culturally appropriate, readable, and suitable for different literacy levels. Although AI models have been shown to improve the readability of patient education materials, further optimization is needed for completeness and linguistic diversity (22).

This review has several limitations. The narrative review approach is useful for synthesizing different types of evidence but is less systematic than a full systematic review and may be more vulnerable to selection bias. Although a structured literature search was undertaken, the narrative methodology may not have identified all relevant publications. Study selection and interpretation involved author judgment and may therefore have introduced subjectivity. The restriction to English language publications could have led to the exclusion of relevant evidence from other settings. The literature was heterogeneous in terms of populations, educational contexts, AI applications and reported outcomes, which limited direct comparison between publications. In addition, some findings and recommendations may need to be updated frequently as artificial intelligence (AI) technologies are evolving rapidly. The proposed framework is conceptual in nature and has not yet been prospectively validated.

Important research gaps remain. Rigorous empirical studies with well-defined objectives, comparators, validated outcomes, long-term follow-up, and transparent reporting are needed to assess AI-based interventions in public health education (17). Research should also include issues of equity, access, algorithmic bias, and impacts on workforce diversity. An inter-disciplinary comparison can reveal specific opportunities and challenges. Longitudinal studies are needed to explore the transfer of AI competencies learned in education to the professional work environment. Another urgent problem is the absence of standard evaluation frameworks for AI-based educational tools (17). Future work should include randomized controlled trials of AI-enhanced educational interventions in public health, longitudinal studies to track competency development over time, and implementation science research to examine barriers and facilitators in varied institutional and geographic settings.

AI is increasingly influencing public health practice and is beginning to enter public health education. Its influence extends beyond technical tasks to the way students learn, analyze data, synthesize evidence, communicate risk, and prepare for population-level decision-making. However, AI will only strengthen public health education if it is integrated responsibly. The evidence base for AI’s educational effectiveness in public health remains limited, and current enthusiasm should be tempered with realistic expectations about what AI can and cannot achieve in educational settings. Public health programs should move beyond *ad hoc* AI use toward structured curricula, competency-based training, faculty development, ethical governance, assessment redesign, and continuous evaluation. The proposed Public Health AI-Education Integration Framework provides a practical model for aligning AI literacy, public health applications, adaptive learning, simulation, human oversight, equity, and academic integrity. However, the framework is proposed as a conceptual model requiring prospective validation through implementation studies, mixed-methods evaluations, and longitudinal tracking of student competency development. Without such validation, it should be viewed as an organizing structure for curriculum development rather than an evidence-based standard. The purpose of AI in public health education is not to replace educators, professional judgment, or community engagement, but to prepare future public health professionals to use AI critically, ethically, and effectively to improve population health. As the World Health Organization has emphasized, AI literacy as a core competency for the next generation of healthcare professionals is essential for navigating the AI-driven public health landscape responsibly (7). The integration of AI into public health education should proceed with caution, guided by evidence, attentive to equity, and grounded in the core values of public health practice.
